# Transcriptome Profile at Different Physiological Stages Reveals Potential Mode for Curly Fleece in Chinese Tan Sheep

**DOI:** 10.1371/journal.pone.0071763

**Published:** 2013-08-26

**Authors:** Xiaolong Kang, Gang Liu, Yufang Liu, Qinqin Xu, Ming Zhang, Meiying Fang

**Affiliations:** 1 Department of Animal Genetics and Breeding, National Engineering Laboratory for Animal Breeding, MOA Laboratory of Animal Genetics and Breeding, College of Animal Science and Technology, China Agricultural University, Beijing, China; 2 College of Agriculture, Ningxia University, Yinchuan, People’s Republic of China; 3 Department of Animal Sciences, Washington State University, Pullman, Washington, United States of America; Huazhong Agricultural University, China

## Abstract

Tan sheep (*Ovis aries*), a Chinese indigenous breed, has special curly fleece after birth, especially at one month old. However, this unique phenotype disappears gradually with age and the underlying reasons of trait evolvement are still unknown. In this study, skin transcriptome data was used to study this issue. In total 51,215 transcripts including described transcripts and transfrags were identified. Pathway analysis of the top 100 most highly expressed transcripts, which included *TCHH* and keratin gene family members, such as *KRT25*, *KRT5*, *KRT71*, *KRT14* and others, showed pathways known to be relevant to hair/fleece development and function. Six hundred differentially expressed (DE) transcripts were detected at two different physiological ages (one-month-old with curly fleece and 48-month-old without curly fleece) and were categorized into three major functional groups: cellular component, molecular function, and biological process. The top six functional categories included cell, cell part, cellular process, binding, intracellular, metabolic process. The detected differentially expressed genes were particularly involved in signal, signal peptide, disulfide bond, glycoprotein and secreted terms, respectively. Further splicing isoform analysis showed that the metallothionein 3 isoform was up-regulated in Tan lamb skin, indicating that it may be related to the conformation of curly fleece in Chinese Tan lamb. The hair-related important differentially expressed genes (*SPINK4*, *FGF21*, *ESRα*, *EphA3*, *NTNG1* and *GPR110*) were confirmed by qPCR analysis. We deduced that the differences existed in expressed transcripts, splice isoforms and GO categories between the two different physiological stages, which might constitute the major reasons for explaining the trait evolvement of curly fleece in Chinese Tan sheep. This study provides some clues for elucidating the molecular mechanism of fleece change with age in Chinese Tan sheep, as well as supplying some potential values for understanding human hair disorder and texture changes.

## Introduction

**Figure 1:**
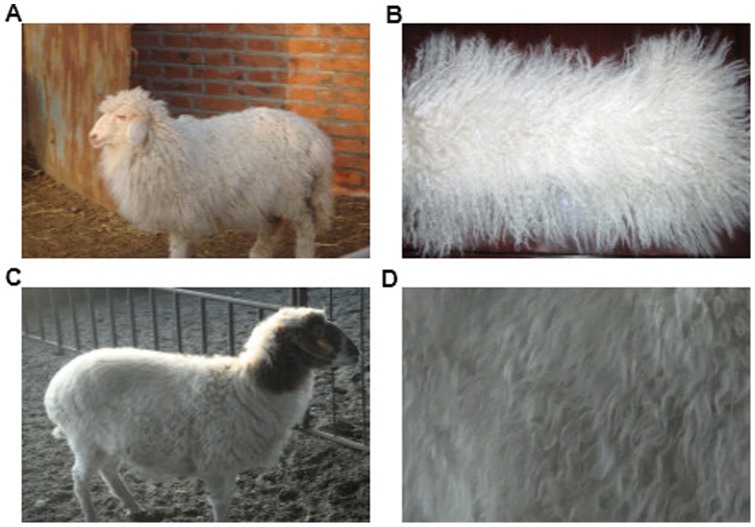
The Chinese Tan sheep and its fleece. A: The Chinese Tan lamb (L); B: The pelt of Tan lamb at one month old; C: The Chinese adult Tan sheep (A); D: The pelt of Tan adult sheep.

Chinese Tan sheep (*Ovis aries*) is one of the most important sheep breeds used for production of high quality pelts in China. It is a short-tailed indigenous sheep breed distributed in northwestern China, such as Ningxia province. Tan sheep are hardy and well adapted to a dry, cold and windy environment. The breed originated from Mongolian sheep, an ancient horned sheep type, and its domestication and breeding resulted in a production of curly fleece. The lamb pelts from Tan sheep are characterized by a natural white color and a lustrous curly fleece. The curly fleece appears when Tan lambs are one month old. After processing, the lamb pelts with curly fleece tend to be thin and light weight, which are well suited for the production of fur coats, carpets, furniture covering, and various forms of handicrafts (Figure 1). However, the curly fleece disappears gradually with age and the mechanisms behind the phenomenon are still unclear.

Early studies related to Chinese Tan sheep mainly focused on genetic evaluation and breed development [1]. Subsequent researchers studied the phenotypic variation between Chinese Tan sheep and other different sheep breeds, including wool color, length, density and shape [2]. In the past decades, various genetic markers were described that characterized the curly fleece of Chinese Tan sheep. Interestingly, a genetic polymorphism of hemoglobin(Hb)was found to be related to wool traits, specifically, the Hb^B^ allele was favorably associated with wool quality [3]. In addition, polymorphisms in the *KRT1.2* (keratin 1.2) and *KAP1.3* (keratin associated protein 1.3) genes were related to the number of wool curvature. Therefore, the researchers concluded that these candidate genes could be used in molecular marker-assisted selection to improve the fleece curvature number of Tan lambs [4]–[5].

Currently, there are several studies that describe the transcriptomes of fetal heart, fetal myofiber and some other tissues in sheep [6]–[8]. However, there is very little transcriptome information related to curly fleece in sheep, except for two studies that examined the cashmere characteristic in goat [9]–[10]. Many different experiments have been carried out in humans and mice that elucidated the formation mechanism of hair texture. These data showed that formation of hair texture is a multistep, complicated process due to many genes involved in multiple key cellular pathways [11]–[12]. Many functional alternations have been detected in related pathways including cell cycles, apoptosis and some other important pathways [13]. To complete understand the complexity of curly fleece formation will require comprehensive cataloguing of gene expression changes at different physiological stages. The objectives of this study, was to use high-throughput sequencing technology to generate comprehensive transcriptome profiles of Tan sheep at two different physiological ages (one-month-old with curly fleece and 48-month-old without curly fleece), and to use this information to investigate the molecular genetic mechanism of its unique curly fleece. This information will identify a repertoire of genes that are expressed in the skin transcriptome and it also aids in the understanding of the development of human hair and texture changes.

## Materials and Methods

### Animal collection and preparation

Experimental procedures were approved by the animal welfare committee of the State Key Laboratory for Agro-biotechnology of China Agricultural University. Twelve unrelated Tan sheep (no common grandparents) at two different physiological stages (one-month-old and 48-month-old) were selected and divided into lamb (L) and adult sheep groups (A). Skin tissue was collected from the shoulder of each sheep after slaughtering and immediately frozen in liquid nitrogen or at −80°C until use.

### RNA extraction, library preparation and sequencing

Trizol^®^ Reagent was used to isolate total RNA from tissues according to the manufacturer’s instructions (Invitrogen, USA). RNA degradation and contamination was assessed on 1% agarose gels. RNA concentration was measured using Qubit^®^ RNA Assay Kit in a Qubit^®^ 2.0 Fluorometer (Life Technologies, CA, USA). RNA purity and integrity was checked using the NanoPhotometer® spectrophotometer (IMPLEN, CA, USA) and the RNA Nano 6000 Assay Kit of the Bioanalyzer 2100 system (Agilent Technologies, CA, USA), respectively.

A total amount of 3 µg RNA was used as input material for the RNA sample preparations. Finally, four samples with RNA integrity number (RIN) values above 8 were used for libraries construction. Sequencing libraries were generated using the IlluminaTruSeq™ RNA Sample Preparation Kit (Illumina, San Diego, USA) following the manufacturer’s recommendations and four index codes were added to attribute sequence to each sample. Briefly, mRNA was purified from total RNA using poly-T oligo-attached magnetic beads. Fragmentation was carried out using divalent cations under elevated temperature in Illumina proprietary fragmentation buffer. First-strand cDNA was synthesized using random oligonucleotides and SuperScript II. Second-strand cDNA synthesis was subsequently performed using DNA polymerase I and RNase H. Remaining overhangs were converted into blunt ends via exonuclease/polymerase activities and enzymes were removed. After adenylation of 3' ends of DNA fragments, Illumina PE adapter oligonucleotides were ligated to prepare for hybridization. In order to select cDNA fragments of 200 bp in length the library fragments were purified with the AMPure XP system (Beckman Coulter, Beverly, USA). DNA fragments with ligated adaptor molecules on both ends were selectively enriched using Illumina PCR Primer Cocktail in a 10 cycle PCR reaction. Products were purified (AMPure XP system) and quantified using the Agilent high sensitivity DNA assay on the Agilent Bioanalyzer 2100 system.

The clustering of index-coded samples was performed on a cBot Cluster Generation System using TruSeq PE Cluster Kit v3-cBot-HS (Illumina) according to the manufacturer’s instructions. After cluster generation, the library preparations were sequenced on an Illumina HiSeq 2000 platform and 90 bp paired-end reads were generated.

### Sequence reads mapping and assembly

Raw data (raw reads) of fastq format were firstly processed through in-house perl scripts. In this step, the clean data (clean reads) were obtained by removing reads containing adapter, reads containing poly-N and low quality reads from raw data. At the same time, quality parameters of clean data including Q20, Q30, GC-content and sequence duplication level were used for data filtering. All the succeeding analyses were carried out using high quality clean data.

Reference genome and gene model annotation files were downloaded from the sheep genome website at http://www.sheephapmap.org/news/OARv2p0.php). The ovine reference sequence from the GenBank database was used for complementary analysis. An index of the reference genome was built using Bowtie v0.12.8 [14] and paired-end clean reads were aligned to the reference genome using TopHat v1.4.0 [15]. TopHat was chosen as the mapping tool because it can generate a database of splice junctions based on the gene model annotation file, and thus give a better mapping result than other non-splice mapping tools. Clean reads were aligned to the reference genome through SOAP2 [16], then duplicated reads and multi-mapped reads were filtered from the alignment results in order to eliminate the PCR (Polymerase Chain Reaction) interference and ambiguous mapping. The clean reads have been submitted to NCBI Short Read Archive under the accession number of SRP018731.

The Cufflinks v1.3.0 [17]–[18] Reference Annotation Based Transcript (RABT) assembly method was used to construct and identify both known and novel transcript fragments (transfrags) from TopHat alignment results. Astalavista v2.2 was used to estimate the five basic alternative splices (ASs) events both in and among the groups based on the results of Cuffmerge and Cuffcompare modules in Cufflinks package [19]. As well differentially expressed (DE) isoforms were estimated by Cufflinks (isoform center).

### Quantification and differential expression analysis of transcripts

HTSeq v0.5.3 (http://www-huber.embl.de/users/anders/HTSeq) was used to count the reads numbers mapped to each transcript. The parameter FPKM (Fragments Per Kilobase of exon per Million fragments mapped) was used to quantify transcripts expression. FPKM was calculated based on the mapped transcript fragments, transcript length and sequencing depth. Currently, this is the most commonly used method for estimating transcript expression [17].

Differential expression analysis of two conditions/groups was performed using the DESeq R package (1.10.1) [20]. DESeq provides statistical routines to determine differential expression in digital gene expression data using a model based on the negative binomial distribution. The resulting P-values were adjusted using the Benjamini and Hochberg’s approach for controlling the false discovery rate [21]. Genes with an adjusted P-value<0.05 found by DESeq were assigned as differentially expressed.

### GO and KEGG enrichment analysis of differentially expressed transcripts

Gene Ontology (GO) enrichment analysis of differentially expressed transcripts was implemented by the GOseq R package [22], in which gene length bias was corrected. GO terms with corrected P-value less than 0.05 were considered significantly enriched by DE transcripts.

KEGG (Kyoto Encyclopedia of Genes and Genomes) is a database resource for understanding functions and utilities of the biological system, such as the cell, the organism and the ecosystem from molecular information, especially for large-scale molecular datasets generated by genome sequencing and other high-throughput experimental technologies (http://www.genome.jp/kegg/). We used KOBAS [23] software to test the statistical enrichment of the top 100 most highly expressed transcripts, DE transcripts and ASs in KEGG pathways, respectively.

### Validation of differentially expressed genes by quantitative real-time PCR

Differentially expressed genes identified by the above described method were validated using quantitative real-time PCR (qPCR). In all cases primers designed for qPCR spanned exon-exon boundaries. *GAPDH* was used as a reference control. Real time PCR was performed using 2× SYBR Green master mix (TianGen) on the CFX96 Real-Time System (BioRAD, USA). The reaction was performed using the following conditions: denaturation at 95°C for 3 min, followed by 40 cycles of amplification (95°C for 30s, 60°C for 30s, and 72°C for 30s). Relative expression was calculated using the delta-delta-Ct method. Primer sequences can be found in Table S1.

## Results

### Identification of expressed transcripts in the sheep skin transcriptome

In this study, 49,647,050 to 60,551,510 raw reads were generated for each sample ( Figure S1 and Table S2). After quality control, 51,215 transcripts (and transfrags) were obtained from the two groups. Of these, 50,856 (99.3%) and 51,119 (99.8%) expressed transcripts were identified in lamb and adult sheep skin, respectively (Table S3), and there were 50,786 commonly expressed transcripts between two groups. The obtained clean transcripts were used for further analysis. Approximately 80% of the total reads were mapped to sheep chromosomes and about 77% of the reads in each sample were uniquely mapped to the sheep genome. The detailed reads density on each chromosome can be found in the supplementary material (Table S4). Chromosomal distribution of the annotated transcripts is shown in Table 1 and Figure S2.

**Table 1:** The genetic information and variation on chromosome based on the skin transcriptome in Tan sheep.

Chromosome	Annotated transcripts	Transfrags	Total No of splice variants
**1**	1540	1558	1154
**2**	1121	1431	878
**3**	1615	1652	1069
**4**	549	571	316
**5**	950	886	638
**6**	412	478	306
**7**	698	640	570
**8**	345	372	226
**9**	350	418	255
**10**	242	362	184
**11**	956	749	702
**12**	488	534	364
**13**	579	576	436
**14**	802	1056	603
**15**	687	514	362
**16**	229	286	152
**17**	385	551	325
**18**	349	553	260
**19**	396	460	339
**20**	546	473	308
**21**	461	378	252
**22**	299	250	207
**23**	212	348	172
**24**	531	558	327
**25**	235	193	193
**26**	171	208	85
**X**	541	477	420
**Total**	15689	16532	11103

**Total No of splice variants: defined as more than one transcript for a ‘genic’ gene. Splice variants denote differences compared with annotated ovine genes.**

The correlation of transcripts expression between samples is the most important indicator for reliability of experimental results and rationality of sampling. Generally, the correlation value should be up to 0.92 (r^2^≥0.92). In our study, the scatter plot showed that the transcripts expression of two biological replicates at each stage (lamb and adult) were similar based on the normalized FPKM values (Figure S3).

**Figure 2:**
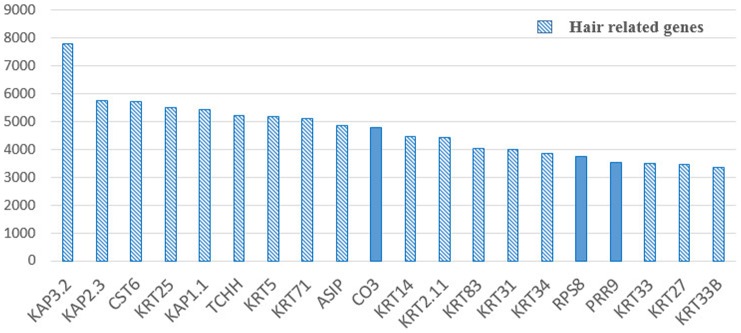
Expression of the top 20 most highly expressed genes in sheep skin. The x-axis shows gene ID; y-axis shows gene expression level (FPKM).

The top 20 annotated transcripts, that ranged from 3,353 to 7,813 FPKM reads (Figure 2) were ranked by abundance and included the keratin protein genes or keratin-associated protein genes: *KAP3.2* (keratin associated protein 3.2), *KAP2.3*, *KRT25* (keratin 25), *KAP1.1*, *KRT5*, *KRT71*, *KRT14*, *KRT2.11*, *KRT83*, *KRT31*, *KRT34*, *KRT33*, *KRT27*, *KRT33B*, and *TCHH* (trichohyalin gene), *RPS8* (ribosomal protein gene), *PRR9* (proline rich 9), cysteine-rich gene, *CST6* (cystatin E/M), *ASIP* (agouti signaling protein) and *CO3* (cytochrome oxidase subunit 3).

### Identification of differentially expressed genes and isoforms between two different physiological development stages

To better survey the biological mechanism of curly fleece, it is important to identify the DE genes between two different stages. There were 600 DE genes that were detected between the two groups when fold changes ≥2 and P<0.05 were used as cutoff values. Further analysis showed that 87 DE genes were significant with strict FDR<0.05 (Table 2, Table S5). Of these, three DE genes were up-regulated while 84 DE genes were down-regulated in lamb skin tissue compared to adult sheep skin tissue.

**Table 2:** Hair-related important DE genes between two groups.

Gene name	Description	Reference
*Wnt2*	Required for repopulation of pigment-producing melanocytes in the hair bulb	[24]
*esrα*(Estrogen receptor α)	Regulates the telogen-anagen follicle transition	[25]
*gpr110*(G protein-coupled receptor 110)	Involved in determining hair texture in humans	[26]
*ephA3* (Ephrin-A3)	Increases the density of hair follicles and accelerates anagen development. Increases proliferation of outer root sheath cells	[27]–[28]
*cyp* (Cytochrome P450)	Participates in the metabolism of endogenous and exogenous substrate metabolism in the skin	[29]
*spink4* (Serine peptidase inhibitor, Kazal type 4)	Member of the Kazal type family of serine protease inhibitors and essential for epithelial tissue homeostasis	[30]
*kap13.1*(Keratin associated protein 13.1)	Related to cashmere traits in goat	[31]
*mitf*(Microphthalmia associated transcription factor)	A newly recognized mediator of Wnt signaling	[32]
*gbp-1* (Guanylate binding protein 1)	Mediator of the anti-proliferative effect of inflammatory cytokines in endothelial cells	[33]

Splice variants have been proposed as a primary driver of the evolution of phenotypic complexity in mammals. In this study, the chromosomal position of each sheep sequence was aligned with the sheep genome and 6,983 and 6,866 splice variants were identified in adult and lamb Tan sheep compared to the annotated sheep genome. Further analysis identified 703 DE isoforms and 492 (69.9%) DE isoforms were annotated. There were 636 and 605 isoforms expressed in adult and lamb skin, respectively. We particularly noticed that isoform metallothionein 3 (*MT3*, CUFF.20060) was up-regulated in Tan lamb skin, and its expression was 4.56755 times (q-value = 0.001173) higher than that in adult sheep (Table S6).

**Figure 3:**
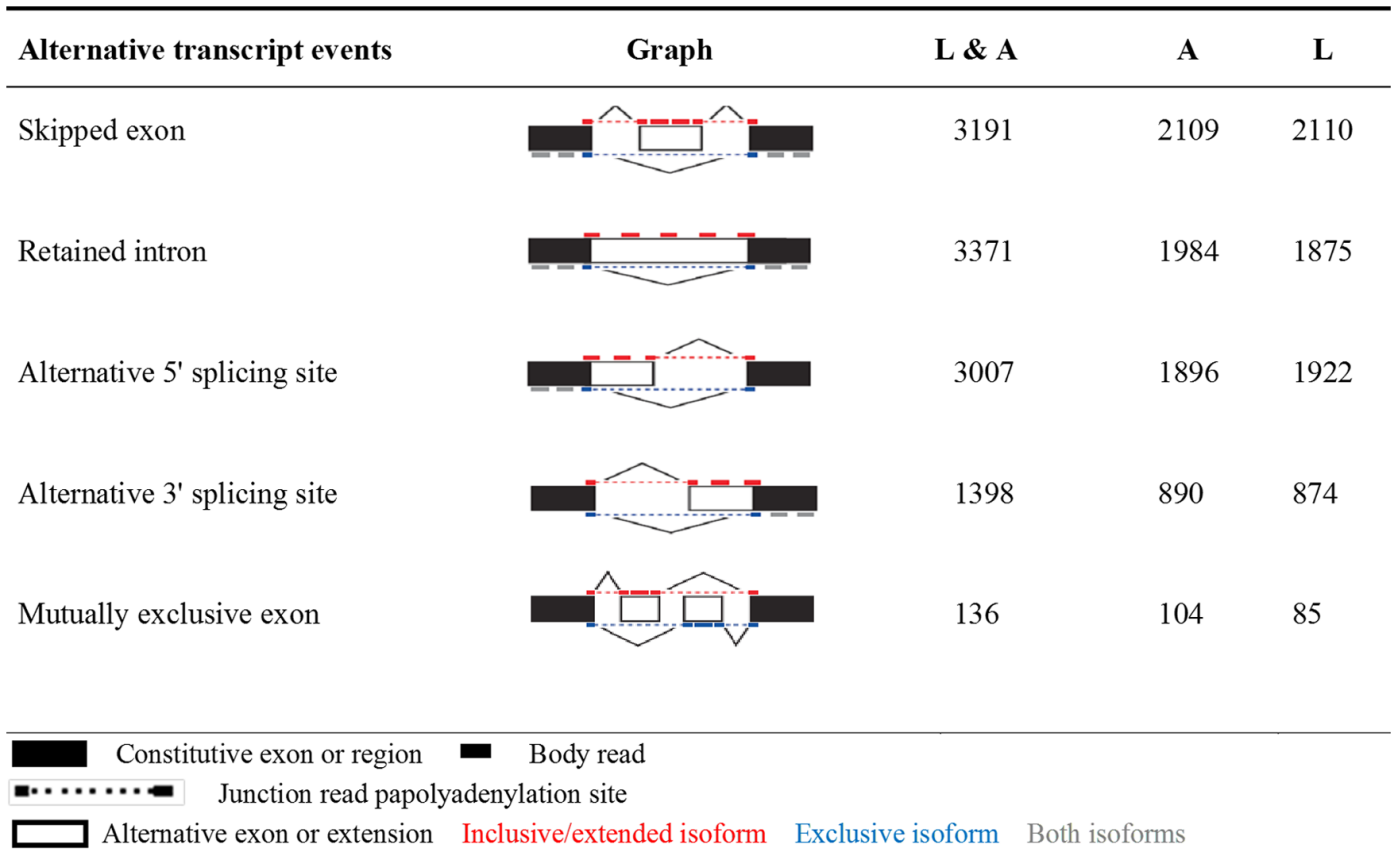
Statistics of mainly alternative splicing events. The first column shows the types of alternative transcript events; the second column shows the splicing graphs and the third to fifth column shows the number of AS events in A and L combined group, A group and L group, respectively.

Additional analysis showed that there were five different splice patterns detected in sheep skin transcriptome data, which included skipped exon (SE), retained intron (RI), alternative 5' splicing site (A5SS), alternative 3' splicing site (A3SS) and mutually exclusive exon (MXE). The first three types, SE, RI and A5SS were the major splicing patterns detected in our study, which represented 86% of the total splicing events; while MXE was a rare event which occurred in only 1.2% of the total events (Figure 3). The number of alternative transcripts distributed on individual chromosome ranged from 85 to 1,154 and averaged 411 splice variants on each chromosome.

### Funtional distribution of differentially expressed genes

**Figure 4:**
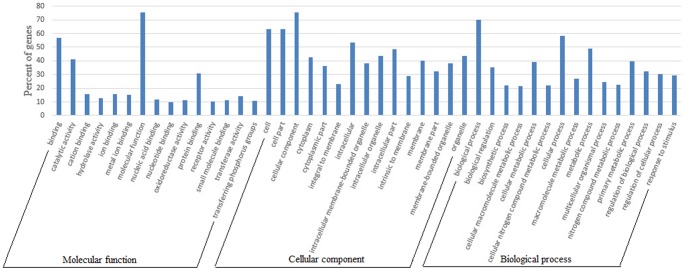
Functional categorization of differentially expressed genes based on known genes in the Uniprot database. The x-axis shows the 2nd level term of Gene Ontology; y-axis shows percent of genes in DE genes.

Differentially expressed genes were considered to be important cause of curly fleece. To better survey the biological behavior of curly fleece, it is necessary to understand the functional distribution of these DE genes in one-month old sheep skin compared to the adult sheep skin. Based on the GO categories a total of 165 clusters were annotated with GO terms. The 600 identified DE genes were categorized into three major functional groups: cellular component, molecular function, and biological process. The abundant genes were categorized into 22 major functional groups (percentage of expressed genes >30) based on the GO categories. The top six functional categories included cell, cell part, cellular process, binding, intracellular, and metabolic process (Figure 4). Further enrichment analysis was related to cellular functions and subcellular locations, and the detected DE genes were enriched in different terms related to hair development. For example, the DE genes *SPINK4*, *FGF21*, *GPR110*, *EphA3* were enriched in signal, signal peptide, disulfide bond, glycoprotein and secreted term, respectively (Table S7).

### Real-time PCR validation of differential gene expression in lamb and adult skin of Tan sheep

**Figure 5:**
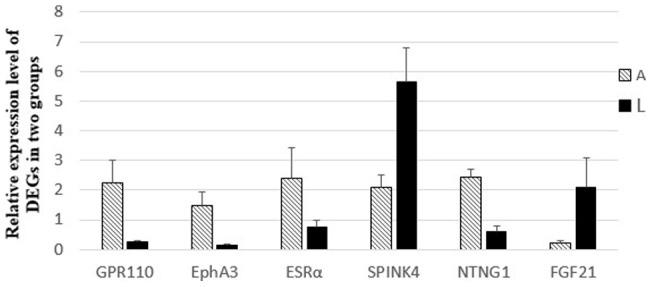
The expression level of differently expressed genes validated by qPCR.

Real-time PCR was used to validate selected differentially expressed genes identified from the RNA-seq data. Six differentially expressed genes (*SPINK4*, *FGF21, ESR*α, *EphA3, NTNG1* and *GPR110*) were selected from the DE genes, which included up- and down- regulated genes between two groups. The results from the real-time PCR confirmed the expression pattern of DE genes at two different stages in Chinese Tan sheep (Figure 5).

## Discussion

Sheep fleece is a distinguishing feature in domestic sheep compared with other farm animals and it is also a hot research topic in animal physiology. On one side, fleece can be processed into goods for people’s lives, such as coat, hat and gloves, etc. On the other side, it is an ideal biomedical model for elucidating the mechanism of hair development in human. The shape and size of fleece has been thought to be determined by the hardening of inner root sheath layers inside the follicle. Large hair follicles produce ‘terminal’ hairs such as those found on the scalp, and curved follicles produce curly hair fibers [34]. However, hair/wool development is a complex process, and the developmental mechanism of fleece between different physiological stages was unclear. In this study, we investigated the formation mechanism of curly fleece in Chinese Tan sheep at two different physiological stages using RNA-seq methods. Our data showed that among the top 20 highly expressed genes in the skin transcriptome 70% (14) of the genes belonged to keratin protein genes, which is consistent with previous reports that the keratin gene family plays an important role in the fleece/hair development [35]. Some of the top 20 expressed genes have previously been verified to play functional roles in hair morphology. For example, the *TCHH* gene is expressed in the developing inner root sheath of the hair follicle, and is associated with hair texture in Europeans [36]. It also plays a structural role within the hair follicle in sheep [37]. *KRT71* (the type II keratin protein), was also reported to be related to hair curliness among species [38]–[39]. All of this evidence suggests that the top genes between two groups may provide some clues to understanding the development of curly fleece in Tan sheep. Further pathway analysis of the top 100 highly expressed transcripts revealed pathways known to be relevant to hair/fleece development and function. Similar to the most highly expressed transcripts, the list of the identified transfrags included transcripts known to be central to hair/wool formation and development. Taken together, these results indicate that we have generated high quality sequence data that is representative of the skin transcriptome in sheep.

Six hundred DE genes were detected between curly fleece (one month old) and non-curly fleece sheep groups (48 months old), which may explain the formation of curly fleece since some important DE genes at the two different physiological stages may participate in hair formation or development. For example, one of the detected DE genes, fibroblast growth factor 21 gene (*FGF 21*), belongs to the *FGF* gene family which function in hair development. Several different studies showed that *FGF* family members (*FGF*1, 2, 5, 7, 10, 13 and 22) were active in mouse skin and their expression changed dynamically in different patterns during the hair growth cycle [40]–[41]. The above information may suggest that *FGF21* also plays a significant role in the regulation of hair growth and related events in Tan sheep. Another detected DE gene, *EphA3* (ephrin A3), a member of ephrins, may act as a hair development promoter [27]. We speculate that *EphA3* may have a potential role in the wool structure of sheep. *WNT2* was also found as a DE gene in the current investigation, which functions in initiating pigmented hair regeneration [24]. Put all together, we conclude that the differentially expressed genes might be important in the formation of different fleece shape at two physiological stages.

The formation of curly fleece is not induced by one pathway, but rather is a result of multiple key cellular pathways that are influenced by many genes. During the formation of curly fleece, involved pathways are mainly composed: *EDA*, *WNT*, *Notch*, *VEGF* and *MAPK* signaling pathways [42]–[47]. The role of the *EDA* pathway in hair follicle biology has been studied and revealed the importance of *EDA* in initiation of hair morphogenesis, hair shaft formation, and hair follicle cycling [43]. *CXCL10* is a functionally important regulation gene in the *EDA* signaling pathway [42]. In our analysis, *CXCL10* was up-regulated in adult skin and activated the *EDA* pathway, which might lead to hair growth and loss of curly fleece in adult skin. *MMP9* (Matrix metallopeptidase 9), another enriched gene in the *EDA* pathway, plays a central role in cell proliferation, migration, differentiation, angiogenesis, apoptosis, host defense, and controls keratinocyte growth. *MMP9* was reported to regulate hair canal formation [44] and may function downstream of *EDA* in some other developmental context [42]. We speculated that both *CXCL10* and *MMP9* have similar functions in hair development, and activated the genes in *EDA* pathway to prompt hair growth and loss of curly fleece in adult sheep. *WNT* signaling molecules play essential roles in many aspects of development, and it is required for the initiation of hair follicle development. In our analysis, the up-regulated low-density lipoprotein receptor-related protein (*LRP*) family activates a conserved “canonical” signaling pathway that causes stabilization of cytoplasmic *β-catenin*, which is an essential regulation factor for hair growth and development. The differentially expressed Dickkopf 1 (*DKK1*) gene, functions by binding and inhibiting *LRP* co-receptors required for activation of canonical *WNT* signaling [47]. Both *LRP* and *DKK1* function on hair growth by activating a conserved “canonical” signaling pathway. Differently expressed isoform analysis showed that the *MT3* isoform was up-regulated in Tan lamb skin, suggesting that *MT3* is related to the conformation of curly fleece in Chinese Tan lamb skin, since the metallothionein 1 was reported to regulate the Menkes kinky-hair syndrome in mouse [48].

Compared with microarray data, RNA-seq generates absolute gene expression measurements with greater resolution and accuracy. These data are then validated by qPCR. Analysis of the transcriptome profile can identify thousands of transfrags variants/isoforms that are expressed in mammalian tissues or organs. In our study, the skin transcriptome greatly accelerated our understanding of gene expression regulation and networks in hair/wool development in Tan sheep at different physiological stages. These data as serve as important resource for revealing the mechanism of genetic variation during Tan sheep fleece development.

## Conclusions

This study has greatly expanded our understanding of the molecular repertoire of the fleece related genes that are involved in the transcriptional response to different physiological stages. Differences were found in expressed genes, splice isoforms and pathways between the two different stages, which constitute the major reasons for explaining the evolvement of curly fleece in Chinese Tan sheep. These results are a valuable resource for biological investigation of fleece evolvement in animals and also supply some potential clues for understanding the molecular mechanisms of human hair development.

## Supporting Information

Figure S1 **Classification of raw reads.** The classification and quality of raw reads from four samples are shown, including clean reads, containing N, low quality, adapter related. Panel A is for L1, B is for L2, C is for A1 and D is for A2, respectively.
(TIF)

Figure S2 **Reads density on chromosomes of the sheep reference genome.** X-axis shows the chromosome position of mapped reads; y-axis, left shows the median of reads density (log_2_), right shows the chromosome number. Panel A is for L1_L2 and panel B is for A1_A2, respectively.
(TIF)

Figure S3 **Correlation plots of the reads for two groups.** X-axis and y-axis shows the log_10_ (FPKM L1_L2) and log_10_ (FPKM A1_A2), separately.
(TIFF)

Table S1 **Primer sequences used in qPCR.**
(DOC)

Table S2 **The data quality analysis in investigated samples.**
(XLSX)

Table S3 **The total transcripts data analyzed in investigated samples.**
(XLSX)

Table S4 **Summary of Illumina sequencing and mapping.**
(XLSX)

Table S5 **Differentially expressed transcripts and isoforms between two groups.**
(XLSX)

Table S6 **Specifically expressed transcripts in investigated groups.**
(DOCX)

Table S7 **Gene Ontology annotation of DE genes between two groups.**
(XLSX)
