# Supplementary figures and images for: Transcriptome Profile at Different Physiological Stages Reveals Potential Mode for Curly Fleece in Chinese Tan Sheep
Source: PLoS One. 2013 Aug 26;8(8):e71763. doi: 10.1371/journal.pone.0071763 (PMC3753335; doi:10.1371/journal.pone.0071763)

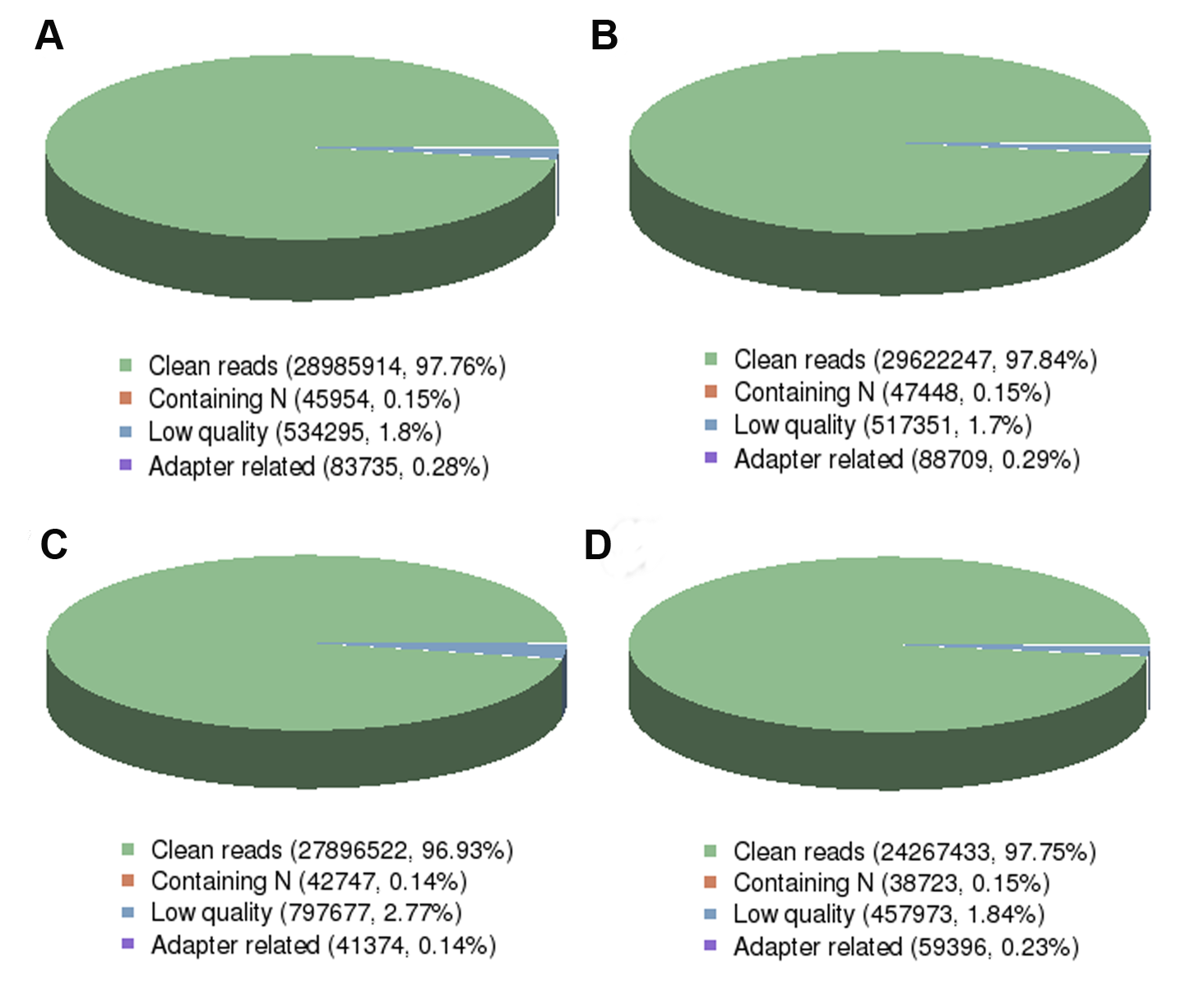

Supplement: Figure S1 — Classification of raw reads. The classification and quality of raw reads from four samples are shown, including clean reads, containing N, low quality, adapter related. Panel A is for L1, B is for L2, C is for A1 and D is for A2, respectively. (TIF) [file pone.0071763.s001.tif]

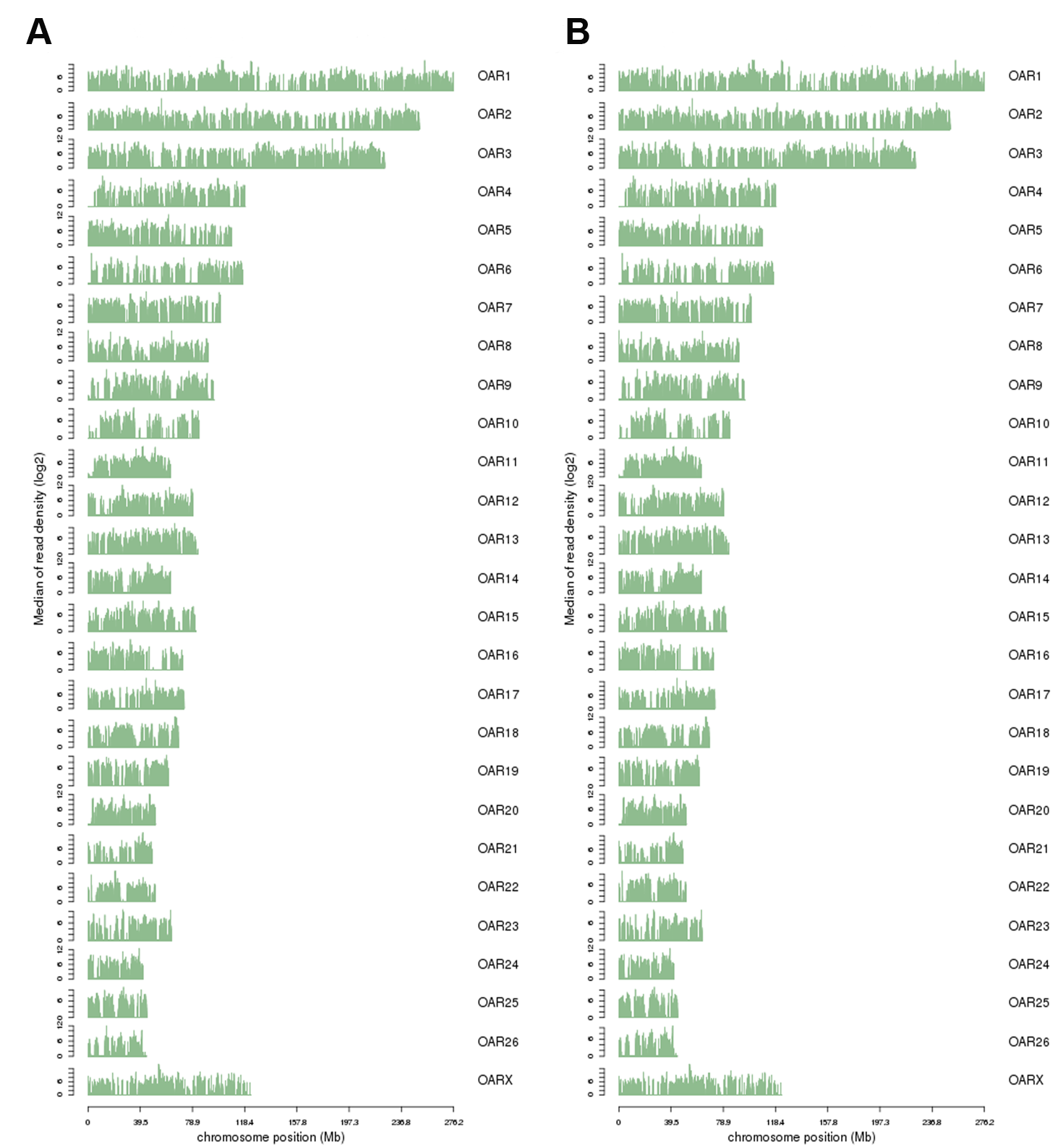

Supplement: Figure S2 — Reads density on chromosomes of the sheep reference genome. X-axis shows the chromosome position of mapped reads; y-axis, left shows the median of reads density (log2), right shows the chromosome number. Panel A is for L1_L2 and panel B is for A1_A2, respectively. (TIF) [file pone.0071763.s002.tif]

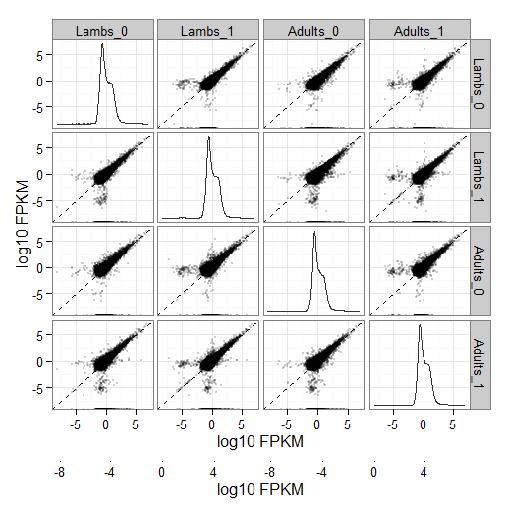

Supplement: Figure S3 — Correlation plots of the reads for two groups. X-axis and y-axis shows the log10 (FPKM L1_L2) and log10 (FPKM A1_A2), separately. (TIFF) [file pone.0071763.s003.tiff]
