# Supplementary material for: Transcriptome Profile at Different Physiological Stages Reveals Potential Mode for Curly Fleece in Chinese Tan Sheep
Source: PLoS One. 2013 Aug 26;8(8):e71763. doi: 10.1371/journal.pone.0071763 (PMC3753335; doi:10.1371/journal.pone.0071763)
Supplement: Table S1 — Primer sequences used in qPCR. (DOC) [file pone.0071763.s004.doc]

**Table S1.** Primer sequences used for quantitative real-time PCR(qPCR)

| Gene Name | **Primers Sequence (5'-3')** | **Size (bp)** | **Tm** |
| --- | --- | --- | --- |
| *EphA3*(ephrin A3) | Forward: CCAGCGATGTATGGAGTTA  Reverse: CTTTCTGCCAGCAGTCTAG | 180 | 60°C |
| *SPINK4*(serine peptidase inhibitor, Kazal type 4) | Forward: TTGTGGACAGGGAGGTG  Reverse: TTATCCGAGCCAAGCAG | 168 | 60°C |
| *GPR110*(G protein-coupled receptor 110) | Forward:CTTGGATAGTAAGCTGCGACA  Reverse: CTTCTGCTTTGATGTCTGGTCC | 80 | 60°C |
| *FGF21* (fibroblast growth factor 21) | Forward: TCCCATTCCAGACTCCAGC  Reverse: CAAAGTGCAGCGATCCGTAC | 263 | 60°C |
| *NTNG1*(NetrinG1) | Forward: CTGTCAATTCATGCCCTT  Reverse: ATCCCAAACTTTCCCTTC | 214 | 60°C |
| *ESRɑ*(estrogen receptor α) | Forward:AGAGAAGTATTCAAGGACATA  Reverse: CTTGTGCTTCAACATTCTCC | 176 | 60°C |
| *GAPDH*(glyceraldehyde-3-phosphate dehydrogenase) | Forward:GTCCGTTGTGGATCTGACCT  Reverse: TGCTGTAGCCGAATTCATTG | 245 | 60°C |

The genes with selection random were identified by real-time PCR. The housekeeping gene, *GAPDH*was taken for internal control. The data was analyzed by C(t) method.
