# Supplementary material for: Transcriptome Profile at Different Physiological Stages Reveals Potential Mode for Curly Fleece in Chinese Tan Sheep
Source: PLoS One. 2013 Aug 26;8(8):e71763. doi: 10.1371/journal.pone.0071763 (PMC3753335; doi:10.1371/journal.pone.0071763)
Supplement: Table S6 — Specifically expressed transcripts in investigated groups. (DOCX) [file pone.0071763.s009.docx]

**Table S6. Annotated and specifically expressed genes and isoforms between two Tan sheep groups**

| **Gene Id** | **Value_1** | **Value_2** | **log2FC** | **p-value** | **q-value** | **Significance** | **Specific expressed gene in two groups** | **Annotation** |
| --- | --- | --- | --- | --- | --- | --- | --- | --- |
| CUFF.13015 | 0.47984 | 4.92318 | 3.35897 | 6.94E-06 | 0.004785 | yes |  | Ovis aries perilipin 1 (PLIN1) |
| CUFF.13015.1 | 0.47984 | 4.92318 | 3.35897 | 6.94E-06 | 0.019113 | yes |  |  |
| CUFF.14017 | 7.06495 | 59.6853 | 3.07863 | 7.96E-07 | 0.000666 | yes | A | Ovis aries interferon alpha-inducible protein 27 |
| CUFF.14017.1 | 7.06495 | 59.6853 | 3.07863 | 7.96E-07 | 0.00409 | yes |  |  |
| CUFF.14527 | 0.198298 | 57.9582 | 8.1912 | 0 | 0 | yes | A | O.aries mRNA for immunoglobulin gamma1 chain secreted form |
| CUFF.14527.1 | 0.198298 | 57.9582 | 8.1912 | 0 | 0 | yes |  |  |
| CUFF.15574 | 2.70149 | 26.0106 | 3.26727 | 1.89E-05 | 0.010397 | yes | A | Ovis aries solute carrier organic anion transporter family, member 1B3 (SLCO1B3) |
| CUFF.15574.1 | 2.70149 | 26.0106 | 3.26727 | 1.89E-05 | 0.039146 | yes |  |  |
| CUFF.16009 | 0.550998 | 24.6342 | 5.48247 | 1.38E-05 | 0.008119 | yes | A | Ovis aries immunoglobulin V lambda chain 5.4.1 gene, partial cds |
| CUFF.16009.1 | 0.550998 | 24.6342 | 5.48247 | 1.38E-05 | 0.030068 | yes |  |  |
| CUFF.16012 | 1.81146 | 249.177 | 7.10387 | 0 | 0 | yes | A | Ovis aries uncharacterized LOC101109564 (LOC101109564) |
| CUFF.16012.1 | 1.81146 | 249.177 | 7.10387 | 0 | 0 | yes |  |  |
| CUFF.17714 | 0.030563 | 0.673638 | 4.4621 | 1.36E-05 | 0.008117 | yes |  | Ovis aries zinc finger protein 33B (ZNF33B) |
| CUFF.17714.1 | 0.030563 | 0.673638 | 4.4621 | 1.36E-05 | 0.030068 | yes |  |  |
| CUFF.19183 | 0.012076 | 1.28768 | 6.7365 | 1.07E-05 | 0.006751 | yes |  | Ovis aries glycine N-phenylacetyltransferase-like |
| CUFF.19183.1 | 0.012076 | 1.28768 | 6.7365 | 1.07E-05 | 0.027721 | yes |  |  |
| CUFF.20060 | 47.5806 | 2.00661 | -4.56755 | 1.42E-07 | 0.000171 | yes | L | Ovis aries metallothionein 3 (MT3) |
| CUFF.20060.1 | 47.5806 | 2.00661 | -4.56755 | 1.42E-07 | 0.001173 | yes |  |  |
| CUFF.22829 | 31.8047 | 290.624 | 3.19184 | 8.60E-07 | 0.000702 | yes |  | Ovis aries phytanoyl-CoA dioxygenase, peroxisomal-like (LOC101107212) |
| CUFF.22829.1 | 31.8047 | 290.624 | 3.19184 | 8.60E-07 | 0.00409 | yes |  |  |
| CUFF.24208 | 0.11543 | 2.74916 | 4.5739 | 3.05E-07 | 0.000319 | yes |  | Ovis aries matrix metallopeptidase 9,MMP9 |
| CUFF.24208.1 | 0.11543 | 2.74916 | 4.5739 | 3.05E-07 | 0.002103 | yes |  |  |
| CUFF.26965 | 0.082558 | 1.59861 | 4.27526 | 9.41E-06 | 0.006059 | yes |  | Ovis aries nitric oxide synthase 2, inducible (NOS2) |
| CUFF.26965.1 | 0.082558 | 1.41186 | 4.09604 | 2.29E-05 | 0.042993 | yes |  |  |
| CUFF.27116 | 0.28219 | 3.79834 | 3.75063 | 1.81E-07 | 0.000209 | yes |  | Ovis aries tumor suppressor candidate 5 (TUSC5) |
| CUFF.27116.1 | 0.28219 | 3.35644 | 3.57219 | 8.91E-07 | 0.00409 | yes |  |  |
| CUFF.27897 | 0.006438 | 15.4512 | 11.2289 | 1.20E-07 | 0.00016 | yes | A | Ovis aries myosin heavy chain 2a (OMYHC2A) |
| CUFF.27897.1 | 0.006438 | 5.61633 | 9.76888 | 5.73E-06 | 0.016914 | yes |  |  |
| CUFF.29248 | 0.048467 | 0.884368 | 4.18958 | 2.08E-05 | 0.011209 | yes |  | Ovis aries protocadherin beta 6 (PCDHB6) |
| CUFF.29248.1 | 0.048467 | 0.884368 | 4.18958 | 2.08E-05 | 0.040856 | yes |  |  |
| CUFF.35115 | 0.022631 | 0.817171 | 5.17424 | 2.04E-06 | 0.00152 | yes |  | Ovis aries guanylate cyclase 2F, retinal, transcript variant 1 (GUCY2F) |
| CUFF.35115.1 | 0.022631 | 0.817171 | 5.17424 | 2.04E-06 | 0.007676 | yes |  |  |
| CUFF.49168 | 0.218502 | 137.965 | 9.30244 | 1.22E-14 | 1.02E-10 | yes | A | Ovis aries major allergen Equ c 1-like (LOC101109939) |
| CUFF.49168.1 | 0.085127 | 31.8635 | 8.54808 | 4.30E-06 | 0.014221 | yes |  |  |
| CUFF.49168.2 | 0.133375 | 84.3934 | 9.3055 | 3.36E-12 | 4.63E-08 | yes |  |  |
| CUFF.56653 | 0.065951 | 2.61251 | 5.30789 | 1.37E-06 | 0.001093 | yes |  | Ovis aries zinc finger protein 33B (ZNF33B) |
| CUFF.56653.1 | 0.065951 | 2.61251 | 5.30789 | 1.37E-06 | 0.005666 | yes |  |  |
| CUFF.56661 | 0.060278 | 3.50632 | 5.86219 | 1.26E-05 | 0.007654 | yes |  | Ovis aries zinc finger protein 33B (ZNF33B) |
| CUFF.56661.1 | 0.060278 | 3.50632 | 5.86219 | 1.26E-05 | 0.030068 | yes |  |  |
| CUFF.58029 | 2.22279 | 53.524 | 4.58974 | 3.91E-11 | 1.31E-07 | yes | A | Ovis aries adiponectin |
| CUFF.58029.1 | 1.65465 | 37.1319 | 4.48806 | 1.09E-08 | 0.000113 | yes |  |  |
| CUFF.8627 | 0.323445 | 3.57631 | 3.46688 | 4.47E-06 | 0.003256 | yes |  | Ovis aries phosphorylase, glycogen, muscle (PYGM) |
| CUFF.8627.1 | 0.323445 | 3.57631 | 3.46688 | 4.47E-06 | 0.014221 | yes |  |  |

Value 1 and value 2 means the value of Tan lamb and adult sheep, separately.
